# De-speckling of medical ultrasound image using metric-optimized knowledge distillation

**DOI:** 10.1038/s41598-025-07115-1

**Published:** 2025-07-03

**Authors:** Mostafa Khalifa, Hanaa M. Hamza, Khalid M. Hosny

**Affiliations:** Department of Information Technology, Faculty of Computers and Informatics, Zagazig University, Zagazig, 44519 Egypt

**Keywords:** Knowledge distillation, Deep learning, Speckle noise, Ultrasound images, Medical research, Mathematics and computing

## Abstract

Ultrasound imaging provides real-time views of internal organs, which are essential for accurate diagnosis and treatment. However, speckle noise, caused by wave interactions with tissues, creates a grainy texture that hides crucial details. This noise varies with image intensity, which limits the effectiveness of traditional denoising methods. We introduce the Metric-Optimized Knowledge Distillation (MK) model, a deep-learning approach that utilizes Knowledge Distillation (KD) for denoising ultrasound images. Our method transfers knowledge from a high-performing teacher network to a smaller student network designed for this task. By leveraging KD, the model removes speckle noise while preserving key anatomical details needed for accurate diagnosis. A key innovation of our paper is the metric-guided training strategy. We achieve this by repeatedly computing evaluation metrics used to assess our model. Incorporating them into the loss function enables the model to reduce noise and enhance image quality optimally. We evaluate our proposed method against state-of-the-art despeckling techniques, including DNCNN and other recent models. The results demonstrate that our approach performs superior noise reduction and image quality preservation, making it a valuable tool for enhancing the diagnostic utility of ultrasound images.

## Introduction

Medical ultrasound imaging plays a vital role in modern healthcare, providing valuable insights into the human body that aid in accurate diagnosis, treatment planning, and monitoring of various diseases and conditions. However, Ultrasound images often suffer from limitations such as Noise, low contrast, and poor resolution, which can hinder healthcare professionals’ ability to extract critical information and make informed decisions. One common type of Noise affecting ultrasound images is speckle noise, which manifests as granular interference and can obscure critical anatomical details^1^. Therefore, there is a growing need for effective image-denoising techniques that can remove speckle noise and improve the quality and interpretability of medical images.

Speckle noise is a multiplicative noise^2^ can be represented using the following equation:

1 $$\:{I}_{n}\left(x,y\right)=I\left(x,y\right)+{I}^{\gamma\:}\left(x,y\right)\mu\:\left(x,y\right)$$

Where I_n_(x, y), I(x, y), and µ(x, y) denote the noisy image, the noise-free image, and the multiplicative speckle noise, µ(x, y) can be simulated using a Gaussian noise with a mean of zero and variance σ, and the value of γ is 1.

In recent years, deep learning has emerged as a powerful tool in medical imaging, demonstrating remarkable success in tasks such as image classification, segmentation, and reconstruction. Presently, convolutional neural network (CNN)-based methods, known for their state-of-the-art performance, have become the primary focus of research. However, these methods often enhance algorithmic performance by increasing the width and depth of neural networks, which in turn result in increased computational demands. Knowledge distillation (KD) techniques have been introduced. To address this issue, knowledge distillation adopts a teacher-student framework that involves transferring knowledge from a larger, more complex model (referred to as the teacher network) to a smaller, more lightweight model (referred to as the student network). By learning from the Teacher’s knowledge, the student network can capture the essence of the data distribution and produce more accurate and meaningful outputs^3^.

This paper proposes a knowledge distillation method for removing speckle noise from ultrasound medical images. Our method aims to leverage the capabilities of CNNs to remove speckle noise from ultrasound images more effectively, while incorporating knowledge distillation techniques to transfer the expertise and diagnostic insights of pre-trained models to the student network. Our approach strives to enhance medical images by improving their sharpness, contrast, and fine details while preserving critical diagnostic features.

We conduct extensive experiments on various medical imaging datasets and evaluate the performance of our proposed method using quantitative and qualitative metrics. The results demonstrate the effectiveness of our approach in enhancing medical images, outperforming existing techniques in terms of image quality and diagnostic accuracy. Our work advances the field of medical imaging by offering an innovative and effective solution for image enhancement. This approach has the potential to improve healthcare outcomes and facilitate more accurate diagnoses.

The key contributions of our study are as follows:

- *Knowledge distillation-based despeckling*: We propose a novel method for speckle noise reduction that combines knowledge distillation with a deep network architecture designed explicitly for ultrasound image despeckling.
- *Optimized training with custom loss*: We develop a custom loss function that utilizes established evaluation metrics (PSNR^4^, SSIM^5^, MSE^6^,) during training. This integrated approach guides the model toward learning patterns that optimize these metrics, resulting in superior denoising outcomes.
- *Benchmarking and improvement*: Our method achieves significant performance improvement compared to existing techniques, demonstrated through PSNR, SSIM, and RMSE^7^, ENL (equivalent number of looks), and FOM evaluations.

The remainder of this paper is organized as follows: Section 2 provides a comprehensive review of related work in medical image enhancement and the application of CNNs and knowledge distillation in this domain. Section 3 presents the proposed methodology, detailing the architecture and training procedure of our KD-based image enhancement framework, as well as describing the experimental setup, including the datasets used. Section 4 outlines the evaluation metrics and presents quantitative and qualitative results. Section 5 presents the results and analysis, providing key insights into the factors that influence the model’s performance, comparisons with other methods, and the limitations of traditional and deep learning-based approaches. The discussion also highlights the robustness of our proposed method, particularly in higher noise conditions, and addresses the challenges posed by large-scale datasets and computational complexity. Finally, Section 6 concludes the paper, summarizing the research’s contributions, limitations, and future directions.

## Related work

**Fig. 1:**
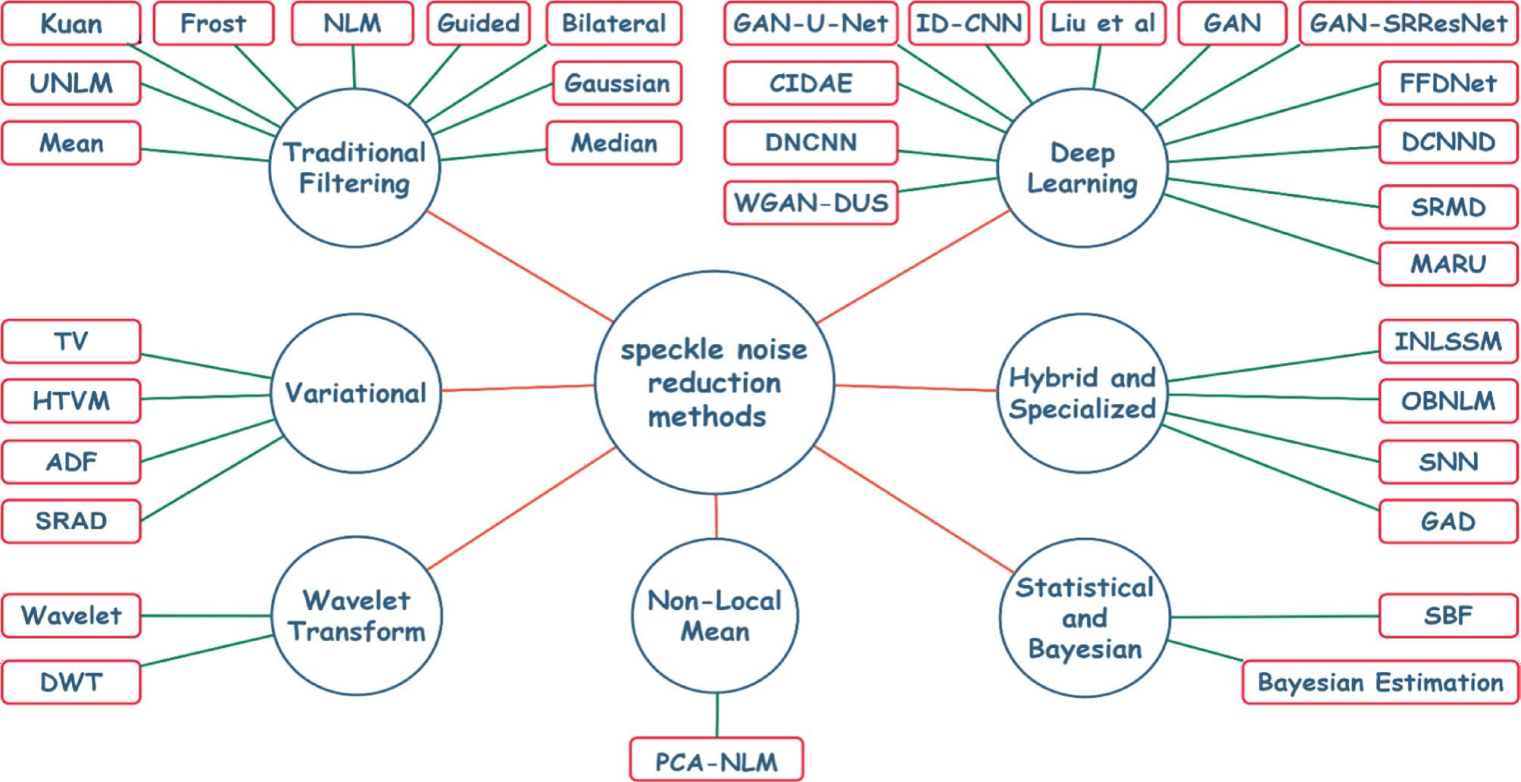
Speckle noise reduction methods.

Medical image enhancement has been extensively researched, with numerous techniques developed to improve image quality and interpretability. Speckle noise reduction methods can be grouped into seven categories based on their underlying principles: traditional filter-based methods, variational methods, transform-based approaches, non-local methods, statistical techniques, hybrid approaches, and deep learning-based methods, as illustrated in Fig. 1:

### Traditional and classical methods

Traditional methods predominantly rely on filtering, where a noisy image is processed using a filter or mask. These methods are straightforward to implement and can reduce speckle noise. However, their primary disadvantage is that they tend to blur edges and image details. Variational methods are effective at preserving edges and essential structures within the image. However, they can be computationally intensive and may introduce artifacts, especially in areas with significant Noise. Transform-based methods differ from spatial filtering approaches by first transforming the noisy image into another domain, such as the frequency or wavelet domain. These methods generally preserve more image details than traditional filters but are more complex and computationally demanding.

Non-local methods achieve high-quality denoising while preserving fine details. The primary disadvantages are their computational expense and slow processing speed, which make them less suitable for real-time applications. Statistical methods adapt to varying noise levels, effectively reducing noise. However, they often require complex parameter tuning and have higher computational demands, which limits their practicality in specific applications. Hybrid methods combine different techniques to leverage the advantages of each. These methods aim to balance the trade-offs between detail preservation, noise reduction, and computational efficiency, though they can be complex to design and implement.

### Deep learning-based methods

Deep learning methods, such as convolutional neural networks (CNNs) and generative adversarial networks (GANs), have demonstrated significant promise in reducing speckle noise. These methods learn complex patterns and features directly from data, providing superior performance compared to traditional techniques. However, they require large amounts of training data and substantial computational power. Our proposed knowledge distillation framework addresses these issues by transferring knowledge from a complex teacher network to a lightweight student network, thereby reducing computational burden while maintaining high performance.

While existing deep-learning methods have achieved remarkable results in reducing speckle noise from medical images, they still face limitations, such as the need for large amounts of annotated training data, which can be scarce due to privacy concerns and the requirement for expert annotations. Additionally, these models often struggle to generalize unseen data and may introduce artifacts or lack clinical relevance. Knowledge distillation techniques were introduced to address these issues. Table 1 provides a comparative overview of existing despeckling methods, highlighting their respective strengths and limitations.

**Table 1:** Comparison of speckle noise reduction Methods.

	References	Functionality/Description	Advantages	Disadvantages
Traditional Filtering	Mean Filtering	Simple averaging filter.	Easy to implement and fast.	Blurs edges and fine details
	Median Filtering^8^	A nonlinear filtering approach that replaces each pixel with the median of its neighbors.	Effective at removing salt-and-pepper Noise, preserves edges.	It can introduce artifacts in the presence of high noise levels.
	Gaussian Filtering^9^	Uses a Gaussian kernel for smoothing	Effectively smooths images and suppresses high-frequency noise.	Blurs edges and details.
	Lee filter^10^	They use local statistics to reduce noise while preserving edges and details adaptively.	Reduces Noise while preserving edges.	It may not be effective in very noisy regions.
	Frost filter^11^	An adaptive filter that reduces speckle noise using an exponential decay function.	Reduces speckle noise while preserving edges.	It may introduce artifacts in homogeneous areas.
	Kuan filter^12^	They adaptively smooth images using MMSE while preserving edges.	Effective at reducing speckle noise.	Computationally intensive.
	Bilateral Filtering^13^	Combines domain and range filtering for edge-preserving smoothing.	Preserves edges while smoothing.	Computationally expensive.
	Guided Filtering^14^	The authors use a guidance image to perform filtering.	Fast, preserves edges.	It may require a good guidance image to be effective.
	Non-Local Means (NLM)^15^	Denoises by averaging similar patches across the image.	Effective at noise reduction, preserves details.	Computationally intensive, slow.
Variational Methods	Total Variation (TV)^16^	Minimizes total variation of the image to reduce noise while preserving edges.	It preserves edges well.	It may produce stair-casing artifacts.
	Anisotropic Diffusion^17^	Smooth images while avoiding blurring edges (Perona-Malik).	Effective at edge-preserving smoothing.	Slow and requires meticulous parameter tuning for optimal performance.
	SRAD^18^	Specific to speckle noise, it reduces noise while preserving edges and details.	Effective for speckle noise, preserve details.	Computationally intensive, slow.
	HTVM (Hybrid Total Variation Model)^19^	Combining features of TV and other models for enhanced denoising	Effective at reducing Noise, preserves edges well.	It may be complex to implement and computationally intensive.
Transform-Based Methods	Wavelet Filtering^20^	It decomposes images into different frequency components and thresholds of wavelet coefficients.	Preserves edges, good at multi-scale noise reduction	It may introduce artifacts if not correctly tuned.
	DWT^21^	Uses discrete wavelet transform for denoising	A good multi-scale representation preserves edges.	High implementation complexity and tuning requirements
Non-Local Methods	PCA-NLM^22^	Combines Principal Component Analysis (PCA) with Non-Local Means (NLM) for enhanced denoising.	Reduces Noise effectively and preserves details.	It is computationally intensive and complex to implement.
Statistical and Bayesian Methods	Bayesian Estimation^23^	Uses probabilistic models to estimate the original image from a noisy observation.	It can be very effective if the model is accurate.	It requires accurate statistical models of noise and signal.
	SBF^24^	Uses statistical approaches for filtering	Effective in various noise conditions.	It may require considerable computational resources.
Hybrid and Specialized Methods	OBNLM^25^	incorporates block matching and various optimization techniques.	Effective, optimized for performance.	Computationally intensive tasks require careful tuning.
	GAD^26^	Adapts based on the local gradient information of the image.	Practical, adapts to image gradients.	Require large computational resources.
	INLSSM^2^	Average pixel values from similar patches to reduce noise.	Effective at leveraging self-similarity.	It is computationally intensive and complex to implement.
	SNN^27^	Speckle Noise Neural Network, specialized for speckle noise.	It is highly effective for speckle noise and preserves details well.	It requires large datasets for training and is computationally expensive.
Deep Learning-Based Methods	CIDAE^28^	Learns to reconstruct the image without Noise.	Effective at learning complex noise patterns.	It requires large datasets for training and may struggle with high noise.
	ID-CNN^29^	Uses an iterative deep CNN approach for denoising	Effective at reducing complex noise patterns.	Requires substantial data and computational resources and multiple training iterations.
	DCNND^30^	Deep CNN for denoising.	High accuracy and effective noise reduction.	May overfit on noise patterns not present in the test data.
	DNCNN^31^	It uses residual learning to denoise images.	It is practical, preserves fine details, and is versatile.	Can struggle with preserving excellent details in extremely noisy images.
	MARU^32^	The enhanced U-Net version improves segmentation and other tasks using multi-scale and residual learning.	Effective at multiple scales, preserves details.	Implementing and optimizing it is challenging due to its multi-scale architecture.
	SRMD^33^	Super-resolution with multiple degradations.	Effective at denoising and super-resolution.	Needs task-specific data and struggles with unseen degradation types.
	FFDNet^34^	The flexible denoising CNN can handle different noise levels.	It is flexible and handles varying noise levels well.	It may require tuning for optimal performance across varying noise levels.
	GAN^35^	Create new data samples through adversarial training.	High-quality results are versatile.	Sensitive to hyperparameters, it can suffer from mode collapse, leading to poor generalization.
	GAN-SRResNet^36^	They use GAN for super-resolution and denoising.	High-quality results preserve details.	Sensitive to adversarial training instability.
	DAGAN^37^	Combines GAN and U-Net for denoising.	Effective, preserves details, robust.	Complex to implement, prone to mode collapse and artifacts.
	WGAN-DUS^38^	It’s a Wasserstein GAN variant addressing mode collapse and training stability issues.	High-quality results and robust training	Careful tuning of the Wasserstein loss and gradient penalty is needed to maintain stability.
	Liu et al.^39^	They used cycle-consistent adversarial networks.	Effective at translating between domains and preserving details.	Complex to implement, requiring careful loss function balancing.

Integrating knowledge distillation with deep learning offers several advantages for speckle noise reduction. It allows for better generalization to unseen data by leveraging knowledge from pre-trained models, even with limited annotated training data. It also helps preserve critical anatomical structures and fine details, enhancing clinical relevance. Moreover, knowledge distillation-based methods can reduce the risk of introducing artifacts or over-smoothing in the despeckled images, resulting in more visually pleasing and diagnostically valuable enhancements.

Understanding the strengths and limitations of each approach through this classification is instrumental in selecting appropriate methods tailored to specific applications. Our proposed knowledge distillation-based method addresses common drawbacks found in existing techniques, presenting a robust solution for enhancing medical images and improving diagnostic accuracy and patient outcomes.

The following sections of this paper present the proposed CNN-based knowledge distillation framework for reducing speckle noise in medical images, including experimental results and discussions, which highlight its effectiveness and advantages over existing approaches. 

## The proposed method

This section describes the methodology of our proposed knowledge distillation framework for despeckling and enhancing medical images. This framework leverages the strengths of Convolutional Neural Networks (CNNs) and knowledge distillation to effectively remove speckle noise, improving image quality while preserving clinical relevance. The key components and steps of our methodology are outlined below.

### Overview

**Fig. 2:**
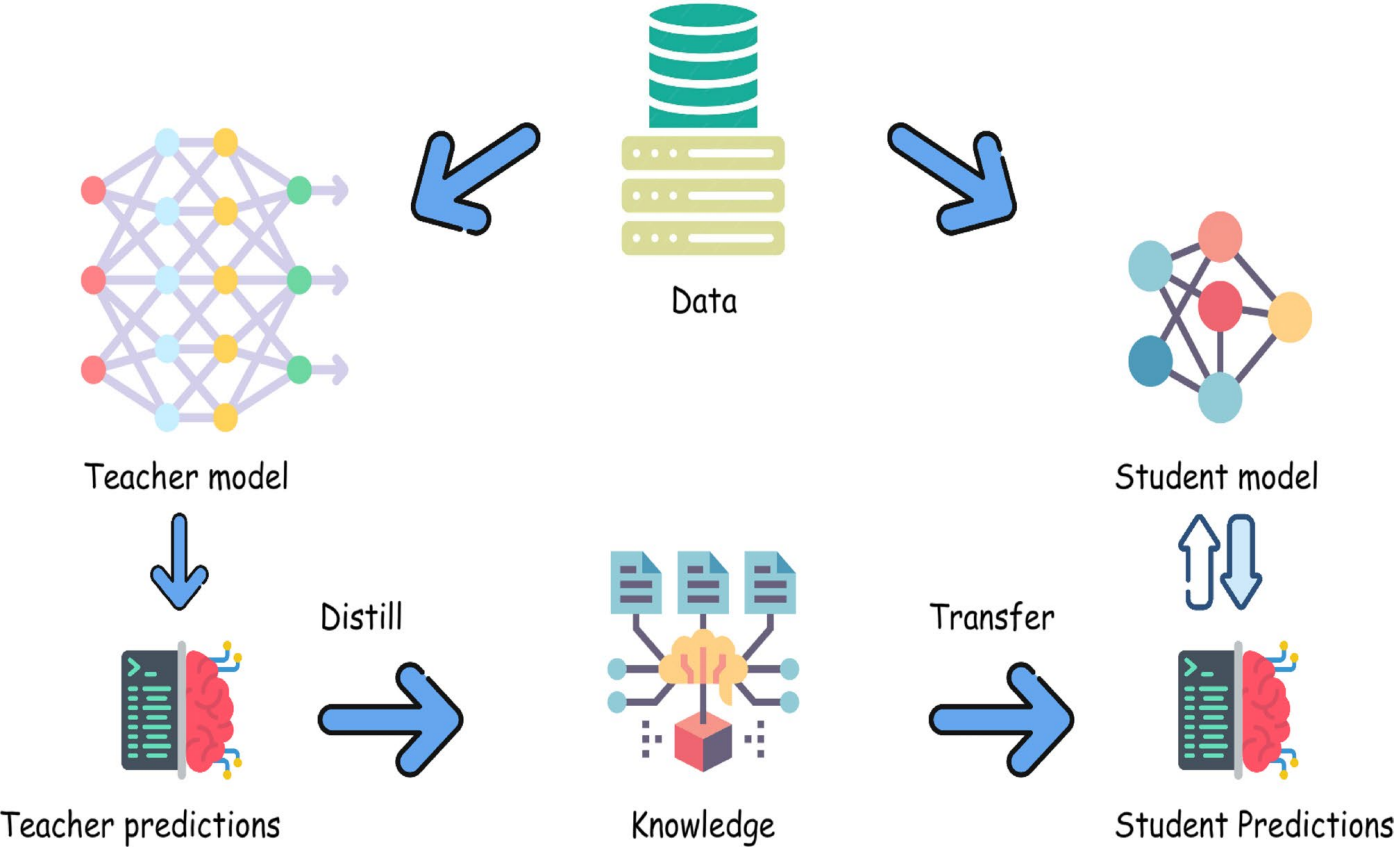
Knowledge distillation framework.

Our methodology uses a knowledge distillation framework to enhance medical images by removing speckle noise. As depicted in Fig. 2, the process starts with a complex teacher network trained on a large dataset to perform high-quality image enhancement. Knowledge is then transferred from this teacher network to a simpler, lightweight student network, which can achieve similar performance with reduced computational requirements. This approach ensures efficient and effective image enhancement, making it suitable for deployment in resource-constrained environments.

The primary motivation for adopting knowledge distillation is to alleviate the computational burden of deep learning models while preserving their efficacy. This is particularly critical in medical imaging, where precise image enhancement is essential for accurate diagnosis and treatment planning. Our framework begins with pretraining a robust teacher network using a diverse dataset. The distilled knowledge is then transferred to a streamlined student network, ensuring it maintains the high standards set by the teacher network while optimizing efficiency.

### Dataset acquisition and preprocessing

We curated a diverse dataset specifically suited for the image enhancement task, focusing on breast cancer diagnosis using the Breast Ultrasound Dataset^40^. This dataset includes ultrasound images from various cases of breast cancer, encompassing both normal and abnormal instances to ensure comprehensive coverage of clinical scenarios. The dataset is representative and balanced, minimizing bias during training and evaluation. It was divided into training, validation, and testing sets; the training set was used to train the knowledge distillation framework, the validation set was utilized for hyperparameter tuning and model selection, and the testing set was reserved for evaluating the final performance of the proposed framework. The study aims to develop a deep-learning model for speckle noise removal using this curated dataset of breast ultrasound images. Speckle noise, a grainy pattern inherent in ultrasound imaging, can hinder accurate diagnosis. By removing this Noise, the model aims to improve the clarity and interpretability of ultrasound images for breast cancer analysis.

The dataset comprises 780 grayscale ultrasound images (average size: 500 × 500 pixels, PNG format) acquired in 2018 from a cohort of 600 female patients aged 25–75. Each image is categorized into one of three classes: normal, benign, or malignant. Medical professionals performed this classification based on established diagnostic criteria. The dataset provides a valuable resource for training and evaluating deep learning models for speckle noise removal, ultimately leading to improved breast cancer diagnosis using ultrasound imaging.

The dataset undergoes preprocessing, including:

- *Normalization*: Pixel values are normalized to zero mean and unit variance for improved training stability and convergence.
- *Resizing*: Images are resized to a consistent resolution for efficient processing.
- *Data Augmentation*: Techniques such as random rotations and flips are applied to increase the diversity of training data.
- Noise Augmentation: To create the training dataset, speckle noise is added to clean images using the multiplicative noise model defined in Eq. 1.
- *Patch Extraction*: The training, validation, and test datasets are divided into smaller patches of 64 × 64 pixels to facilitate batch processing. This approach reduces GPU requirements and enhances learning efficiency by focusing on localized image features.

### Network architecture

**Fig. 3:**
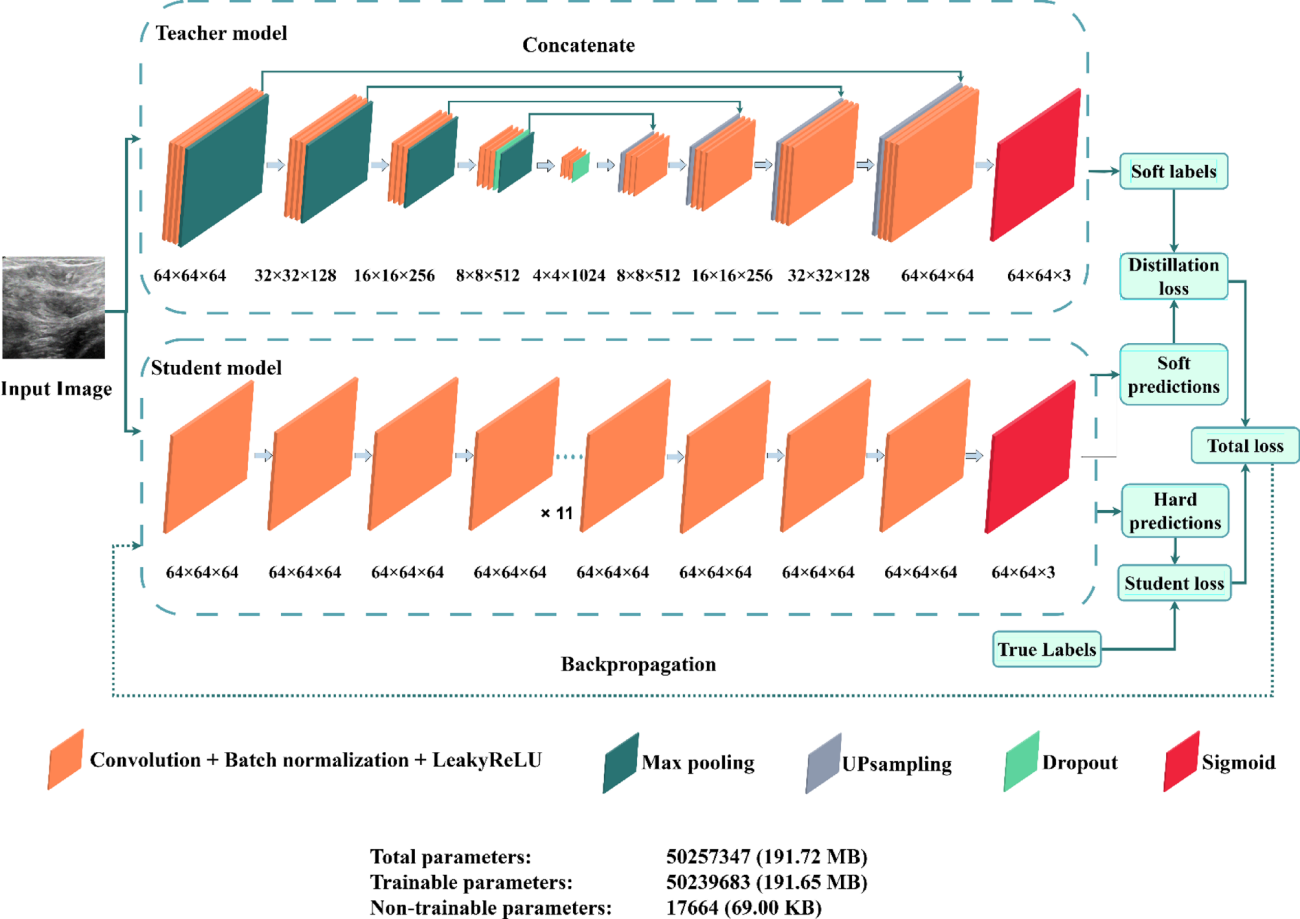
The proposed network architecture.

In this subsection, we describe the architectural design of both the Teacher and student networks used in our framework, as represented in Fig. 3. The teacher network is selected based on its proven effectiveness in image enhancement tasks, typically employing a complex U-Net architecture. In contrast, the student network is designed to be lightweight and computationally efficient. The architectural choices focus on simplifying the model while ensuring the student network can effectively capture essential features and patterns in medical images, enabling faster inference and deployment in real-world scenarios.

#### The teacher network

As depicted in the upper section of Fig. 3, the Teacher model is a deep convolutional neural network (CNN) engineered to perform comprehensive feature extraction and refinement for speckle noise reduction in medical images, particularly ultrasound scans. This model is pivotal in guiding the student model by providing high-quality, denoised outputs that serve as reference points during training.

##### Input processing

The Teacher model begins by processing the input image, which typically has dimensions of 64 × 64. The input first passes through an initial convolutional block, which comprises three fundamental operations:

- *Convolution*: The convolutional layers apply a series of filters (64 in total) to the input image, capturing various spatial features, including edges, textures, and patterns. These layers are crucial for transforming the raw pixel values into feature maps that represent different levels of abstraction.
- *Batch Normalization*: Following convolution, batch normalization helps stabilize and accelerate training. By normalizing the activations, batch normalization helps mitigate the internal covariate shift, leading to more robust and faster convergence.
- *LeakyReLU Activation*: The LeakyReLU (Leaky Rectified Linear Unit) activation function introduces non-linearity into the model, allowing it to learn more complex patterns. Unlike the standard ReLU, LeakyReLU prevents neurons from becoming inactive by allowing a slight, non-zero gradient when the input is negative.

##### Encoding path

The encoding path of the Teacher model is designed to progressively reduce the spatial dimensions of the feature maps while increasing the depth, or the number of filters. This path comprises several convolutional blocks, each followed by max-pooling layers, which reduce the spatial dimensions (e.g., from 64 × 64 to 32 × 32, and so forth). The max-pooling layers help the network capture high-level, abstract features by down-sampling the feature maps, effectively condensing the information, and making the model invariant to small translations of the input image.

##### Bottleneck

At the center of the Teacher model, a bottleneck structure is implemented using a series of convolutional layers with very high depth (e.g., 1024 filters) but small spatial dimensions (e.g., 4 × 4). The bottleneck is a critical component, concentrating the most salient features necessary for speckle noise reduction. It acts as a compressed input representation, containing only the most essential information while discarding irrelevant details, which allows the model to focus on the specific patterns associated with Noise and its reduction.

##### Decoding path

The decoding path reconstructs the feature maps to the original spatial dimensions (64 × 64), producing a denoised version of the input image. This path mirrors the encoding path, with upsampling layers replacing the max-pooling layers. By interpolating the feature maps, the upsampling layers increase the spatial dimensions (e.g., from 4 × 4 to 8 × 8, 16 × 16, etc.). Convolutional layers that refine the features at each resolution are interleaved with these upsampling operations.

An essential aspect of the decoding path is the use of skip connections. These connections link corresponding layers in the encoding and decoding paths, allowing the model to combine features from different resolutions. This strategy helps preserve spatial details that might be lost during the down-sampling process.

The Teacher model concludes with a final convolutional layer that outputs a denoised image with dimensions 64 × 64 × 3, representing the RGB channels of the cleaned image. This output is the reference or “soft label” for the student model in the knowledge distillation process.

##### Advantages of U-Net for despeckling

1. *Encoder-Decoder Structure*: This architecture effectively captures and processes complex features in noisy images, allowing the model to distinguish and remove Noise while reconstructing clean images.
2. *Skip Connections*: These connections help preserve important spatial details by merging high-resolution features from the encoder with the abstract features in the decoder, which is crucial for maintaining fine details in despeckled images.
3. *Flexibility*: The model’s U-Net-like structure allows it to be trained end-to-end on pairs of noisy and clean images, enabling it to adapt to various noise types and imaging conditions. This makes it versatile and practical in different medical imaging applications.

These advantages make the provided model highly effective for reducing speckle noise. The architecture is designed to denoise images and preserve essential image details, making it a robust tool for enhancing the quality of medical images, such as ultrasound scans.

#### Student network

The student model, as depicted in the lower section of Fig. 3, is a streamlined and computationally efficient version of the Teacher model. Its primary role is to replicate the performance of the Teacher model while using significantly fewer resources, making it suitable for deployment in environments with limited computational capacity.

##### Input processing and network structure

Like the Teacher model, the student model begins by processing the input image through convolutional operations. However, the student model’s architecture is simplified to reduce the number of parameters and computational load.

##### Convolutional blocks

The student model employs a series of identical convolutional blocks, each consisting of convolution, batch normalization, and LeakyReLU activation functions, like the Teacher model. Instead of progressively reducing spatial dimensions and increasing depth, the student model uses a uniform structure with Convolutional Blocks repeated 16 times. This setup ensures consistent feature extraction throughout the network. This design offers a robust learning process, striking a balance between effective feature extraction and strong prediction capabilities.

##### Network depth and parameter efficiency

The student model does not include a bottleneck layer like the teacher model. Instead, it maintains a relatively shallow architecture, processing the image through multiple layers without significantly reducing the spatial dimensions or increasing the channel depth. This design ensures that the model remains lightweight and efficient, suitable for constrained computational resources.

##### Output and knowledge transfer

The student model ultimately produces an output with the exact dimensions as the Teacher model’s output (64 × 64 × 3), representing the denoised image. During training, the student model learns to approximate the Teacher model’s performance through knowledge distillation. The loss function used in this process combines the discrepancy between the student’s output and the Teacher’s soft labels (distillation loss) and the difference between the student’s output and the actual labels (student loss).

By leveraging the distilled knowledge from the Teacher model, the student model achieves high performance in speckle noise reduction while being more efficient and faster to deploy in real-time applications.

### Loss function

The loss function is crucial in guiding the learning process of both the Teacher and student networks in our framework. It balances noise reduction, image enhancement, and knowledge transfer objectives. We utilize a combination of MSE, SSIM, and L1 loss^41^., and distillation loss^42^ to achieve optimal performance. Each type of loss contributes uniquely to the overall learning process, ensuring that the enhanced images maintain high fidelity and clinical relevance.

#### The loss function for the teacher network

The loss function for the teacher network is designed to achieve high-quality image enhancement results. This network is typically pre-trained on a large dataset and uses a combination of content and perceptual losses to ensure that the output images retain essential structural and contextual information. The teacher network’s performance is crucial, as it serves as the benchmark for the student network during the knowledge distillation process.

#### The loss function for student network

The loss function for the student network is designed to facilitate effective knowledge transfer from the teacher network. In addition to the standard content and perceptual losses, we incorporate a distillation loss that aligns the student network’s outputs with those of the teacher network. This loss function encourages the student network to learn from the Teacher’s expertise, enhancing its performance while maintaining a lightweight architecture suitable for deployment on resource-constrained devices.

To effectively guide the learning process of our student network, we designed a custom loss function that balances multiple objectives, including noise reduction, image enhancement, and knowledge transfer. The custom loss function integrates Mean Squared Error (MSE), Structural Similarity Index (SSIM), L1 loss, Peak Signal-to-Noise Ratio (PSNR), and a distillation loss. These components enforce the denoised images’ reconstruction accuracy, perceptual similarity, and pixel-level fidelity. The formulation of the custom loss function is detailed as follows:

- *L1 Loss*^41^ The mean absolute error (MAE) is calculated between the true and predicted images. L1 loss encourages sparsity and reduces overall absolute differences. It ensures that the denoised images remain close to the actual images, adds robustness against outliers, and enhances edge preservation.

2 $$\:L1\:Loss=\frac{1}{MN}\sum\:_{x=1}^{M}\:\sum\:_{y=1}^{N}\:|\text{g}\left(x,y\right)-p\left(x,y\right)|$$

where g(x, y) and p_s_(x, y)​ are the ground truth and predicted images, respectively.

- *Knowledge distillation loss*^42^ measures the discrepancy between the Teacher’s and the student’s predictions, scaled by a temperature parameter to soften the logits. It ensures that the student network effectively mimics the teacher network’s performance, thereby enhancing its learning process and overall performance. The distillation loss is computed as:

3 $$\text{Distillation Loss}=\text{K}\text{L}\left(\text{S}\text{o}\text{f}\text{t}\text{m}\text{a}\text{x}\left({p}_{\text{t}}/T\right)\parallel\:\text{S}\text{o}\text{f}\text{t}\text{m}\text{a}\text{x}\left({p}_{\text{s}}/T\right)\right)$$

Where p_t_​ is the Teacher-predicted images, p_s_​ is the student-predicted images, and KL divergence KL(P∣∣Q) measures the divergence of the probability distribution P from the distribution Q.

The total loss function is then formulated as follows:

4 $$\:\text{Total Loss}=\frac{\text{MSE}+\alpha\:\cdot\:\text{Distillation Loss}+\beta\:\cdot\text{SSIM}+\gamma\:\cdot\:\text{L1 Loss}}{\text{PSNR}}$$

where α, β, and γ are weights assigned to the respective loss components to balance their contributions. In our implementation, we set the distillation weight α = 0.3, β = 1, temperature T = 0.8, and a small epsilon ϵ=1 × 10^−10^ to prevent division by zero in the distillation loss calculation.

The knowledge distillation process is guided by the distillation loss, which represents the link between the teacher network and student network. The teacher model learns complex feature representation for image despeckling and provides a feature map, which is then converted into probability using the softmax function. But instead of directly applying the softmax function, we use a softened version of the probability distribution by dividing the teacher and student logits by the temperature parameter. Then, every time through the process, knowledge is taken from the teacher network by reducing the difference between the feature maps of the teacher and student networks. This is called distillation loss.

Temperature is the main parameter that controls the transfer of knowledge, modulating the softmax function to maintain the sharpness of the probability distribution generated by a teacher model. This, in turn, influences the student’s learning process. Let’s describe how temperature works:

- *T = 1*: The softmax function will behave normally, emphasizing the highest probability class and producing a sharp distribution. That model will ignore classes with a low probability of having speckle noise.
- *T > 1*: This produces a softer probability distribution that includes more information about secondary class predictions.
- *T < 1*: The softmax function becomes sharper. Producing high-confidence predictions.

We set t = 0.8, which enhances the teacher’s confidence in identifying noisy regions while retaining some softer information, thereby improving the student model’s ability to balance noise suppression with the preservation of structural details.

We integrate SSIM and PSNR into our loss function to ensure that the evaluation metrics directly affect the cost function at each iteration of the model’s operation. By doing so, the model receives continuous feedback from its output, enabling it to adjust its coefficients iteratively. This approach effectively reduces speckle noise while preserving crucial image details, as evidenced by high SSIM and PSNR values, which indicate better structural similarity and signal-to-noise ratio. By incorporating these metrics into the loss function, we ensure that the student network prioritizes reducing speckle noise while maintaining high image quality. This feedback mechanism enables the model to optimize its performance dynamically, striking a balance between noise reduction and preserving image details. This comprehensive loss function framework ensures that the student network effectively learns to produce high-quality denoised images while integrating the knowledge distilled from the teacher network.

Since PSNR is our primary evaluation metric, we include it in the loss function not as a separate term, but as a normalizing factor. Unlike SSIM, MSE, L1, and distillation loss, which measure errors or differences, PSNR reflects the overall quality of the restored image, with higher values meaning better output. Because PSNR values are usually larger than the other losses, dividing the total loss by PSNR helps normalize the scale and encourages the model to increase PSNR while minimizing the other losses.

This setup differs from standard loss functions, which combine the components. By dividing the total loss by PSNR, the model is guided to produce results with higher perceptual quality, not just lower pixel-level errors. This structure encourages the network to favor outputs that both minimize noise and achieve higher PSNR, which is directly linked to improved visual quality. Our experiments, including the results presented in Table 4 and the ablation study in Section 4.4, demonstrate that this strategy enables the model to achieve improved performance across all key evaluation metrics.

Another critical point is that these losses provide a balance between sharpness and smoothness. While MSE causes the image to be oversmoothed, SSIM helps to retain structural details. Combining these losses ensures that the final image maintains a balance between noise suppression, structure preservation, and perceptual quality.

### The training procedure

We integrate knowledge distillation into our training process by aligning the feature representations of the student network with those of the teacher network, which involves fine-tuning the student network using a distillation loss that encourages it to mimic the teacher network’s outputs on a subset of the training data. This process facilitates the transfer of knowledge from the more complex teacher network to the simpler student network, thereby enhancing its performance and generalization capabilities. The training procedure includes several critical steps:

- The Model Architecture and Initialization:
  - Network Architectures: The teacher and student network architectures are selected and designed to suit the image enhancement task.
  - Weight Initialization: Both networks are initialized with appropriate weights and biases to ensure adequate training.
- Optimizer: The Adam optimizer utilizes its adaptive learning rate and momentum for efficient optimization of model parameters.
- Validation: A separate validation set is used for regular evaluation to monitor training progress and adjust hyperparameters as needed.
- Custom Loss Function: The custom loss function combines Mean Squared Error (MSE), Structural Similarity Index (SSIM), distillation Loss, L1 loss, and the PSNR to achieve comprehensive model optimization. Additionally, a distillation loss term encourages the student network to mimic the teacher network’s outputs on a subset of the training data, facilitating knowledge transfer and enhancing performance.
- Callbacks and Monitoring: Callbacks, such as learning rate reduction, are employed to optimize training efficiency and ensure consistent performance improvement.
- Training Iteration: The training process iteratively optimizes the networks’ parameters, monitoring loss values and convergence to ensure effective learning.

The training process was meticulously configured with careful attention to various hyperparameters. The learning rate was initially set at 0.0005 and adjusted throughout training using learning rate decay or adaptive learning rate methods. A batch size of 32 was chosen to strike a balance between training stability and memory requirements, considering both memory capacity and computational efficiency.

Training iterations were limited to 100 due to the convergence of loss functions and diminishing returns in performance improvement. A learning rate reduction callback was used to track progress and avoid overfitting, which lowered the learning rate if the iteration PSNR did not improve. The training was performed on GPU-accelerated hardware with memory growth enabled to handle the model’s computational needs efficiently.

To optimize model performance, we carefully tuned hyperparameters, including learning rate, batch size, and regularization techniques. Experiments were conducted in a Kaggle environment with a 16GB GPU to accelerate training. Cross-validation was employed to prevent overfitting and ensure robust performance evaluation.

This research developed a knowledge distillation framework for enhancing medical ultrasound images, as outlined in Algorithm 1. A pre-trained teacher model effectively transferred knowledge to a compact student model, implemented using TensorFlow. Leveraging Kaggle’s resources, the framework efficiently handled the complex dataset and learned from noisy input-output pairs. Guided by the teacher network, this advanced deep-learning approach yielded a robust denoising algorithm that significantly improves image quality for practical applications.

**Algorithm 1:**
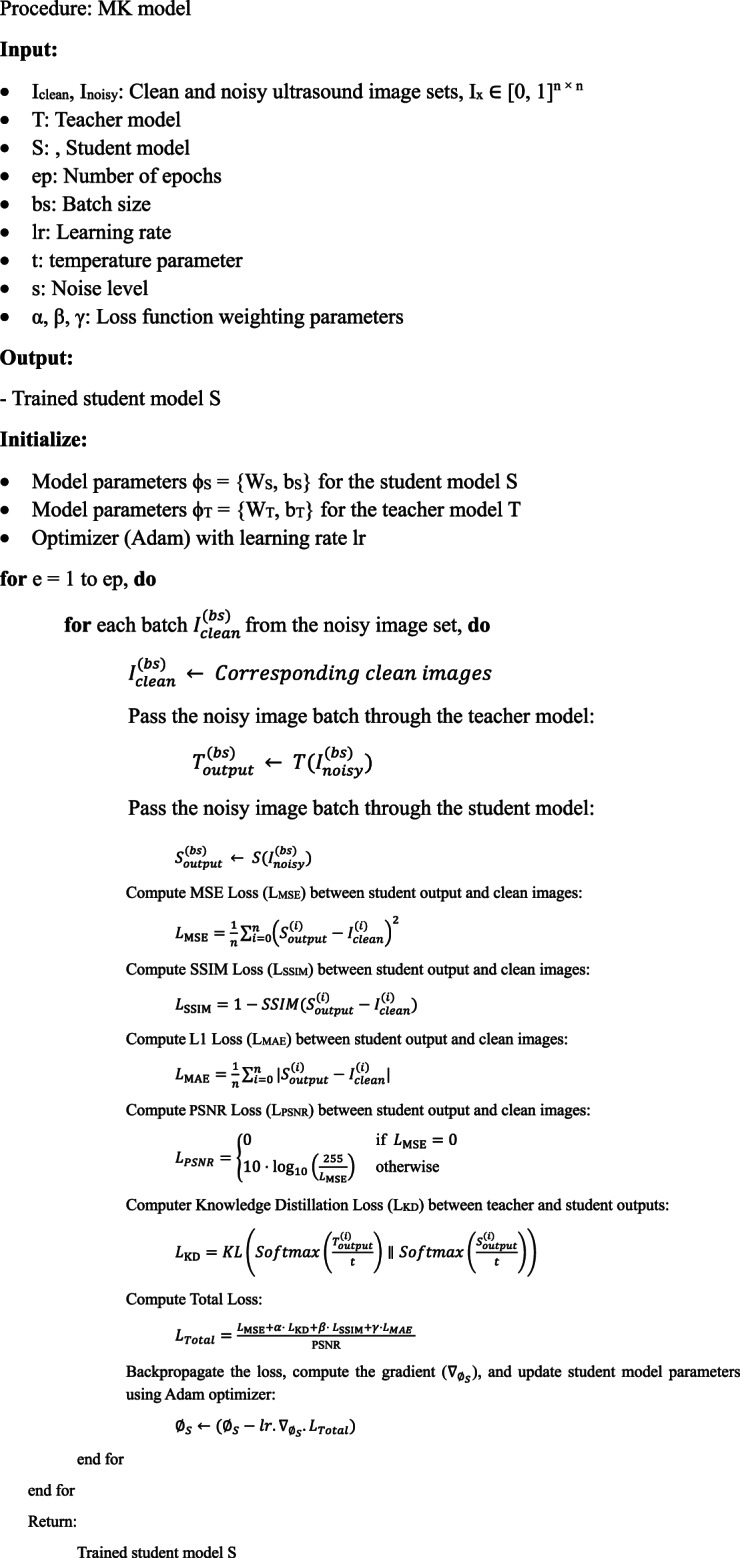
The proposed MK model.

## Results and analysis

This section presents the results and analysis of experiments evaluating the performance of our proposed model for medical image enhancement. We compare our model’s performance with baseline methods and state-of-the-art quantitative and qualitative approaches. Relevant tables, figures, and visualizations support our findings, offering an in-depth analysis of the results. We discuss the strengths and limitations of our approach, share insights gained from the experiments, and consider potential factors influencing the model’s performance.

### Evaluation metrics

Comprehensive quantitative and qualitative assessments were conducted to evaluate the performance of the proposed framework. A quantitative evaluation was performed using standard metrics, including PSNR and SSIM, which provided objective measures of image quality, fidelity, and similarity to the ground truth. We also compared the performance of our framework with that of existing speckle noise reduction methods to highlight the improvements made by our approach.

- *Mean Squared Error (MSE)*: Measures the average squared difference between the predicted and ground-truth images, ensuring accurate reconstruction at the pixel level.
- *Structural Similarity Index Measure (SSIM)*: Evaluates the structural similarity between predicted and ground truth images. The use of SSIM ensures that the denoised images not only match the ground truth structurally but also maintain the perceptual quality and details of the images.

5 $$\:\text{S}\text{S}\text{I}\text{M}=\frac{\left(2{\mu\:}_{x}{\mu\:}_{y}+{c}_{1}\right)\left(2{\sigma\:}_{xy}+{c}_{2}\right)}{\left({\mu\:}_{x}^{2}+{\mu\:}_{y}^{2}+{c}_{1}\right)\left({\sigma\:}_{x}^{2}+{\sigma\:}_{y}^{2}+{c}_{2}\right)}$$

- *Peak Signal-to-Noise Ratio (PSNR)*: Quantifies the quality of the denoised images relative to the original images. It is derived from the MSE and is used to scale the total loss. A higher PSNR indicates better image quality and lower total loss. Therefore, the loss function computation divides all other losses by the PSNR value.

6 $$\:\text{P}\text{S}\text{N}\text{R}=\left\{\begin{array}{ll}0&\:\text{if MSE}=0\\\:10\cdot\:{\text{l}\text{o}\text{g}}_{10}\left(\frac{255}{\text{M}\text{S}\text{E}}\right)&\:\text{otherwise}\end{array}\right.$$

### Quantitative results

We measured our model’s performance using established quantitative metrics such as PSNR and SSIM. We compared these metrics with those obtained from baseline methods and state-of-the-art approaches on the same dataset. The results indicate that our model outperforms the baseline methods and achieves competitive performance compared to the state-of-the-art approaches. Tables 2 and 3 summarize the quantitative results, showcasing the improvements achieved by our model in terms of PSNR, SSIM, RMSE, ENL, and FOM values.

**Table 2:** PSNR, SSIM, and RMSE values for images under different noise variances.

Denoising method	PSNR (dB)			SSIM			RMSE		
	0.01	0.09	0.25	0.01	0.09	0.25	0.01	0.09	0.25
Average	26.051	18.835	15.203	0.668	0.346	0.213	0.049	0.114	0.174
Median^8^	26.271	18.771	15.106	0.680	0.346	0.215	0.048	0.115	0.176
Gaussian^9^	27.761	19.782	16.063	0.779	0.494	0.378	0.041	0.103	0.157
Bilateral^13^	25.071	18.575	15.048	0.592	0.295	0.170	0.056	0.118	0.177
CIDAE^28^	28.013	18.988	15.141	0.870	0.404	0.225	0.051	0.112	0.174
DNCNN^31^	48.482	36.990	29.320	0.992	0.907	0.792	**0.004**	**0.008**	0.024
Proposed	**49.221**	**39.032**	**33.913**	**0.997**	**0.953**	**0.872**	**0.004**	0.013	**0.023**

**Table 3:** ENL and FOM values for images under different noise variances.

Denoising method	ENL			FOM		
	0.01	0.09	0.25	0.01	0.09	0.25
Average	2.754	2.760	2.792	0.953	0.891	0.837
Median^8^	2.719	2.674	2.551	0.954	0.891	0.836
Gaussian^9^	2.695	2.678	2.673	0.961	0.903	0.853
Bilateral^13^	2.820	2.840	2.891	0.951	0.891	0.837
CIDAE^28^	2.592	2.576	2.820	0.957	0.895	0.839
DNCNN^31^	3.146	3.149	3.287	0.996	0.994	**0.988**
Proposed	**9.103**	**9.193**	**9.503**	**0.997**	**0.987**	0.977

### Qualitative results

**Fig. 4:**
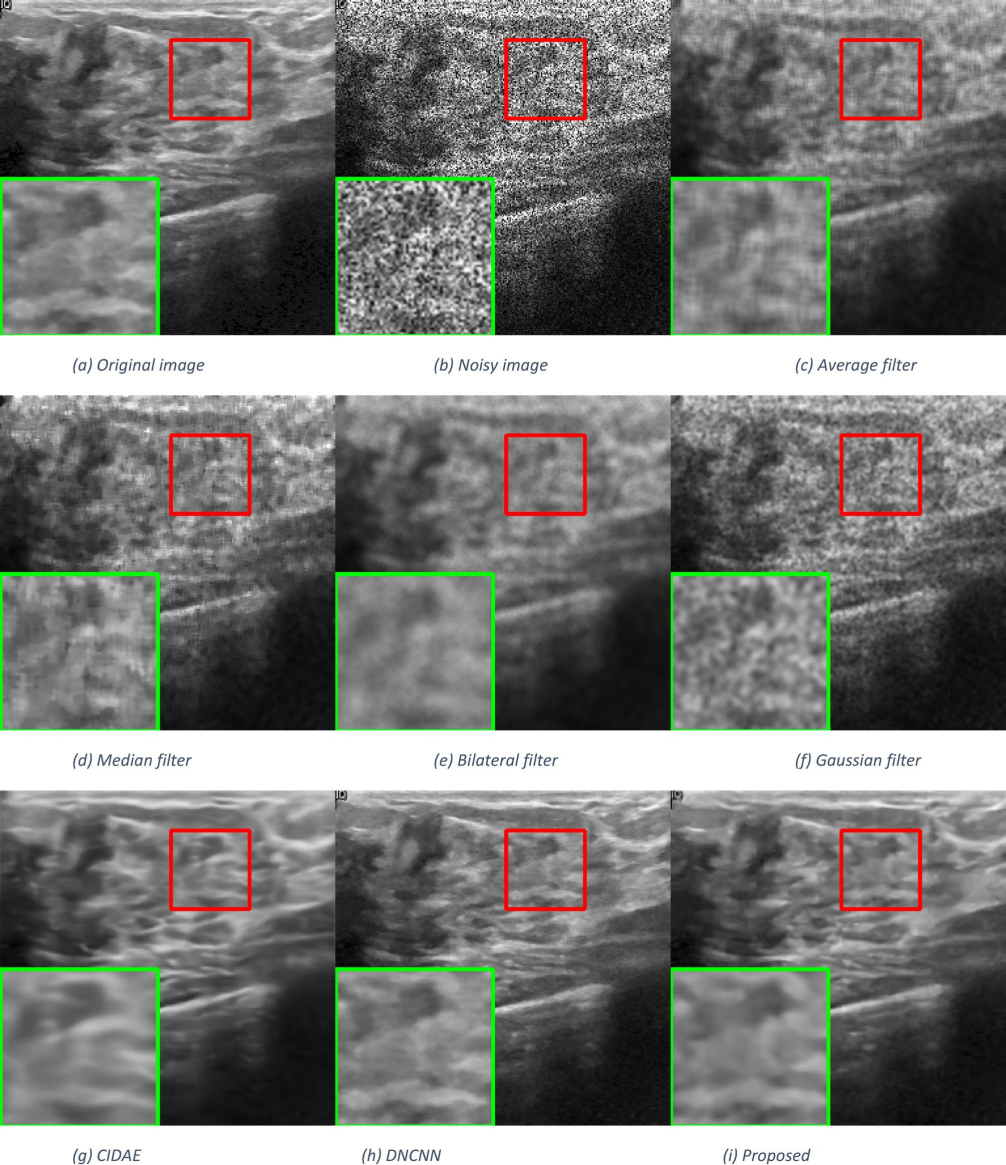
Qualitative comparisons of the proposed method with other despeckling methods on a breast ultrasound image at noise standard deviation σ = 0.25.

**Fig. 5:**
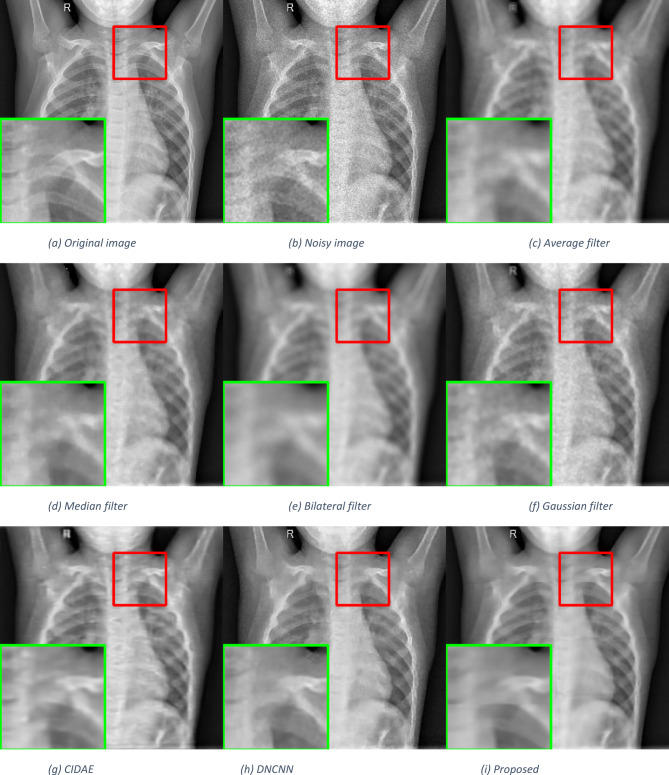
Qualitative comparisons of the proposed method with other despeckling methods on a chest ultrasound image^43^.

**Fig. 6:**
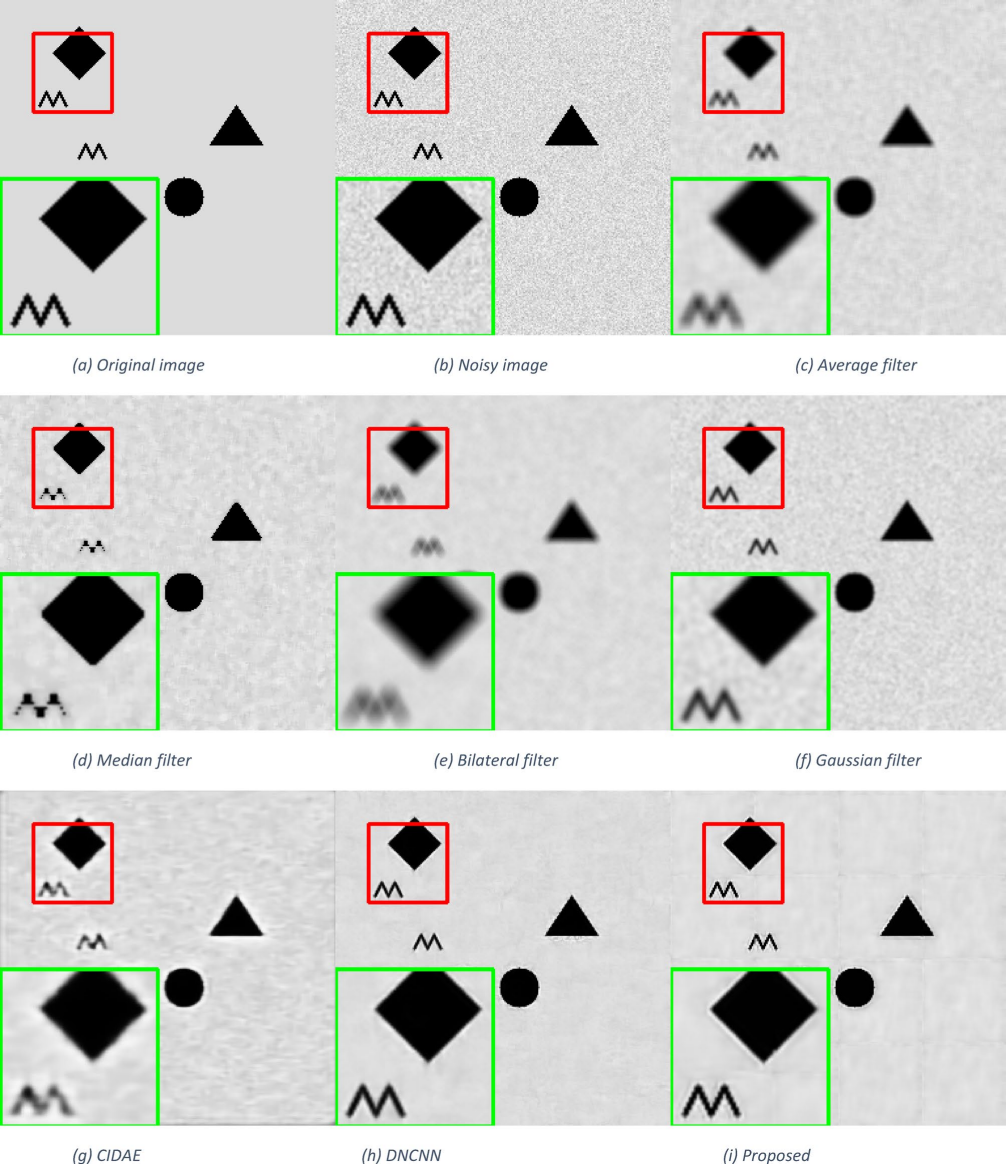
Qualitative comparisons of the proposed method with other despeckling methods on a synthetic image.

To evaluate the visual quality of the enhanced images, we conducted qualitative assessments using images from the Breast Ultrasound dataset, the Chest X-Ray Images (Pneumonia) dataset^43^, and a set of synthetic images. We compare the images generated by our model with those produced by the baseline methods and state-of-the-art approaches. Figures 4, 5 and 6 showcase sample images, including the original unenhanced images and those enhanced by different methods. Visual inspection reveals that our model enhances image quality by improving clarity, contrast, and noise artifacts.

The average filter effectively removes speckle noise but significantly loses image details due to the substantial blurring of edges and essential structures. The median filter reduces noise more effectively than the average filter and preserves edges better, but it still causes some blurring, especially in regions with refined details or sharp transitions. Conversely, the bilateral filter performs poorly, resulting in a blurrier image than any other approach. The Gaussian filter performs better than the average, median, and bilateral filters in preserving tiny features while blurring the image.

Deep learning-based techniques offer superior performance compared to traditional methods, but some, like CIDAE, may produce lower performance due to noticeable blurring in most image areas. On the other hand, DNCNN delivers significantly higher performance than all of the previous algorithms in that it reduces speckle noise and generates a more precise image. Additionally, delicate structures and texture details are better retained. The proposed method offers the best balance between noise reduction and detail preservation, maintaining edges and textures with minimal blurring.

### Ablation study

**Fig. 7:**
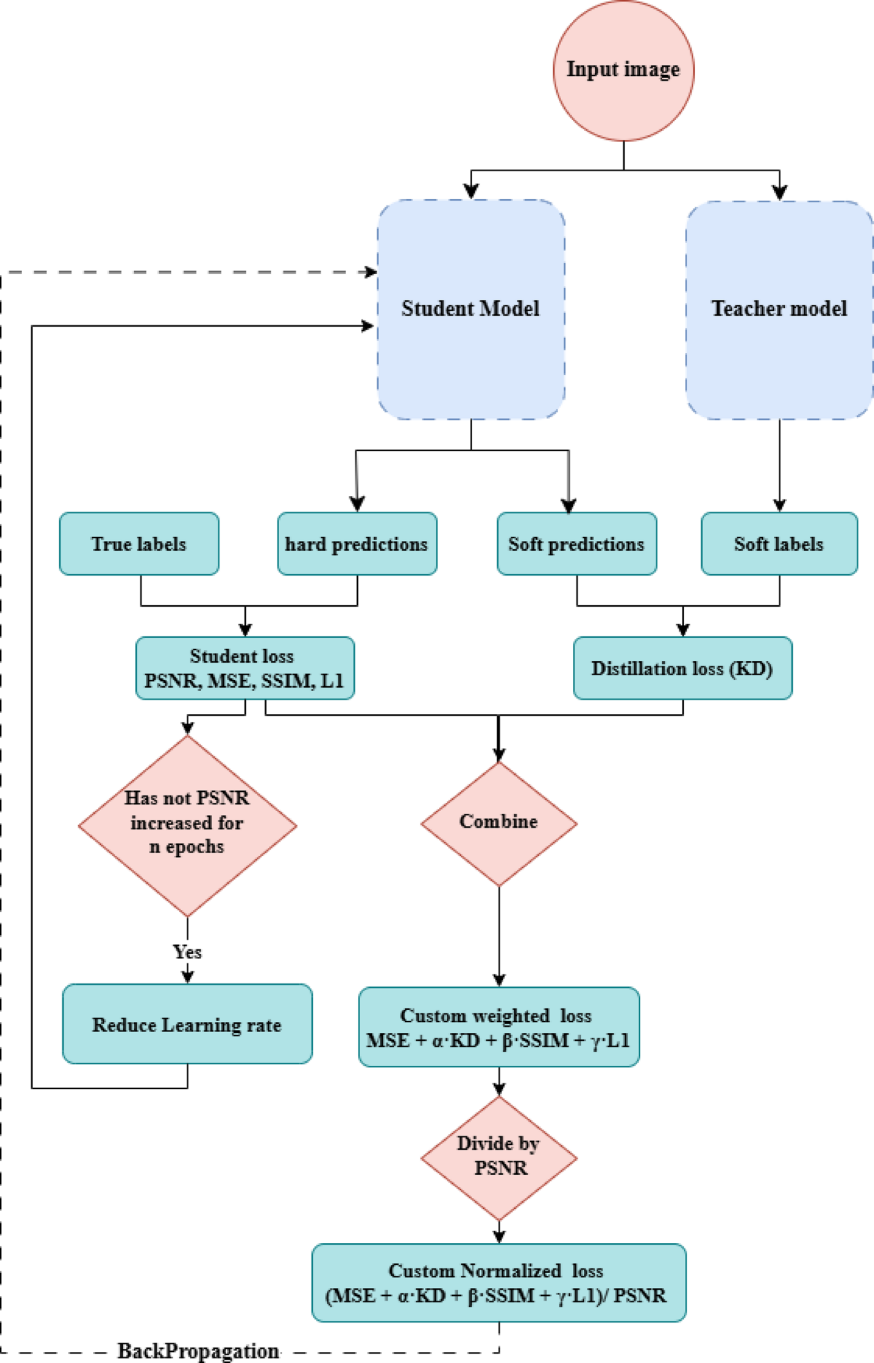
Proposed Deep Learning Training Pipeline with PSNR-Normalized Loss and Adaptive Learning Rate Scheduling.

To demonstrate the effectiveness of the MK model and the novelty of the proposed loss function, we perform a series of ablation experiments on the breast ultrasound dataset. These experiments include both quantitative and qualitative results to highlight the impact of key components. As shown in Fig. 7, two main factors significantly influence the performance of our model: the normalized loss function and the learning rate reduction strategy.

*The effectiveness of learning rate reduction function.* We evaluate the effectiveness of the learning rate reduction function by testing two different strategies. First, we implement Exponential Decay, which automatically reduces the learning rate after a specified number of steps. Next, we use the PSNR value from the evaluation metrics to control the learning rate: if the PSNR does not improve after a certain number of model iterations, the learning rate is reduced to maximize the resulting PSNR and improve image accuracy. Table 4 illustrates the impact of using ReduceLROnPlateau compared to a fixed learning rate schedule. As observed, our model achieves significantly better PSNR, SSIM, and RMSE values when using ReduceLROnPlateau, even with the removal of certain loss function components.

*The effectiveness of normalized loss function.* In image restoration tasks, it is common to combine multiple losses (such as MSE, L1, SSIM, and KD) through weighted summation to balance pixel fidelity, perceptual quality, and feature preservation. However, these approaches treat the loss as static, regardless of the output quality. In contrast, we propose a dynamic loss modulation strategy by normalizing the total loss with the PSNR of the current output. Specifically, the total loss is computed as (MSE + α·KD + β·SSIM + γ·L1)/PSNR. This design ensures that when the output quality is low (i.e., PSNR is low), the effective loss becomes larger, enforcing stronger gradients and faster correction. As the output improves (i.e., PSNR increases), the effective loss magnitude naturally decreases, encouraging finer adjustments and preventing overfitting. To the best of our knowledge, PSNR-based dynamic normalization within the loss function has not been previously explored for despeckling or related image restoration tasks. Our experiments demonstrate that this formulation leads to better convergence behavior, improved PSNR, SSIM, and RSME scores, and more stable training compared to traditional weighted-sum losses.

As shown in Table 4, introducing PSNR normalization into the loss formulation improves the PSNR by approximately 1.7 dB and SSIM by 0.002 compared to standard summation-based losses, confirming its effectiveness.

**Table 4:** Effect of PSNR-Normalized loss vs. Standard loss formulations and learning rate strategies on breast ultrasound dataset when σ = 0.01.

	ReduceLROnPlateau			Fixed learning Rate schedule		
Loss Formulation	PSNR (dB) ↑	SSIM ↑	RMSE ↓	PSNR (dB) ↑	SSIM ↑	RMSE ↓
α·KD + β·SSIM + γ·L1	47.6434	0.9949	0.0047	47.4049	0.9950	0.0048
MSE + β·SSIM + γ·L1	44.7988	0.9926	0.0064	46.2602	0.**9968**	0.0056
MSE + α·KD + γ·L1	47.0218	0.9949	0.0048	46.7821	0.9942	0.0051
MSE + α·KD + β·SSIM	44.1536	0.9937	0.0067	39.7874	0.9922	0.0121
MSE + α·KD + β·SSIM + γ·L1	47.5124	0.9962	0.0049	43.8301	0.9909	0.0069
**(MSE + α·KD + β·SSIM + γ·L1)/PSNR (Ours)**	**49.2210**	**0.9970**	**0.0040**	**47.4854**	0.9956	**0.0047**

We also conduct experiments to investigate the two key factors controlling the knowledge distillation process: the distillation weight (λ) and temperature. We tested various values, and Table 5 summarizes some of the samples we tried, including distillation weights of 0.2, 0.3, and 0.4, and temperature values of 0.7, 0.8, and 0.9. As shown in Table 5, our model produces the best metrics when the distillation weight is 0.3 and the temperature is 0.8.

**Fig. 8:**
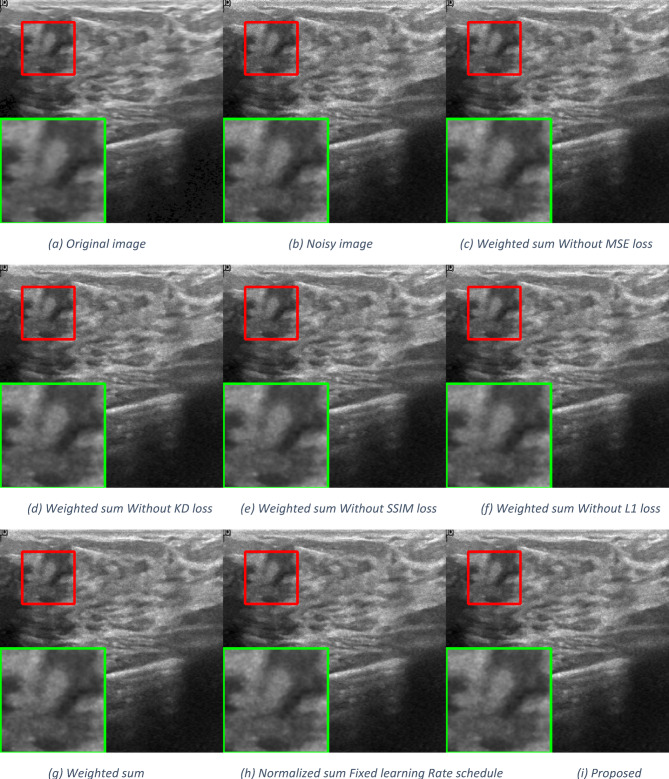
Impact of PSNR-Normalized Loss, Standard Loss, and Learning Rate Reduction on the Breast Ultrasound Dataset (σ = 0.01).

Finally, Fig. 8 summarizes the impact of using the normalized loss function combined with learning rate reduction. This approach encourages the model to maximize the PSNR value, resulting in images that are both visually appealing and closest to the ground truth.

**Table 5:** Performance comparison with distillation weights and temperature values (σ = 0.01).

Distillation Weight (λ)	Temperature (T)	PSNR (dB)	SSIM	RMSE
**0.2**	0.8	47.7053	0.9961	0.0045
**0.3**	0.8	**49.2210**	**0.9970**	**0.0040**
**0.4**	0.8	48.0281	0.9953	0.0044
**0.3**	0.7	47.3804	0.9941	0.0048
**0.3**	0.9	47.9941	0.9959	0.0044

## Discussion

Our experiments gave us valuable insights into the factors influencing the model’s performance. The quality and diversity of the training dataset significantly impact the model’s ability to generalize across different medical imaging scenarios. Hyperparameters such as learning rate, batch size, and network architecture are crucial for optimizing performance. Furthermore, pre-trained models or transfer learning techniques can enhance results by leveraging prior knowledge from related tasks or domains. Importantly, the choice of the cost function directly affects the model’s performance, guiding the optimization process.

The results demonstrate that the proposed method significantly outperforms other methods, including traditional and deep learning-based approaches, across varying Noise levels (σ values). The proposed method’s consistent superiority in PSNR and SSIM underscores its effectiveness in image-denoising tasks.

- *Low Noise (σ = 0.01)*: The proposed method achieves a PSNR of 49.221 dB at this noise level and an SSIM of 0.997. It surpasses the DNCNN method (a CNN-based approach) with a PSNR of 48.682 dB and an SSIM of 0.992. The CIDAE method, based on autoencoder architectures, also lags with a PSNR of 28.013 dB and an SSIM of 0.870, which shows that while deep learning methods like DNCNN and CIDAE are effective, the proposed method introduces further refinements that improve performance, even at low noise levels.
- *Moderate Noise (σ = 0.09)*: When Noise increases to σ = 0.09, the proposed method maintains a strong lead with a PSNR of 39.0032 dB and an SSIM of 0.953, which is considerably better than DNCNN, which achieves a PSNR of 36.990 dB and an SSIM of 0.907, and CIDAE, which reaches 18.988 dB PSNR and 0.404 SSIM. The performance gap suggests that the proposed method’s architecture is remarkably robust against moderate noise, handling it better than the autoencoder-based CIDAE and the CNN-based DNCNN.
- *High Noise (σ = 0.25)*: At the highest noise level tested (σ = 0.25), the proposed method’s robustness is particularly evident, achieving a PSNR of 33.913 dB and an SSIM of 0.872, which is significantly higher than DNCNN’s PSNR of 29.320 dB and SSIM of 0.792. CIDAE, with a PSNR of 15.141 dB and an SSIM of 0.225, struggles significantly in these conditions. The performance of traditional methods like Gaussian and Bilateral filtering is notably poor at this level, further emphasizing the advantages of deep learning approaches in handling high noise levels.

The proposed method performs much better in ENL than in all other methods, but its FOM and RMSE values are more closely aligned with the DNCNN approach.

### Key insights

- *Robustness of the Proposed Method:* The proposed method consistently outperforms traditional and advanced deep learning-based methods across all noise levels. Its superior performance, especially in higher noise conditions, suggests that the method’s architecture is highly effective at preserving image quality.
- *Comparison of Deep Learning-Based Methods:* While DNCNN and CIDAE both leverage deep learning techniques—CNNs and autoencoders, respectively—they fall short compared to the proposed method. DNCNN, while competitive, shows a more significant performance drop at higher noise levels compared to the proposed method, indicating potential areas for improvement in its architecture or training process.
- *Limitations of Traditional Filtering:* Traditional filtering methods like Gaussian and Bilateral lag behind deep learning-based methods, particularly at higher noise levels. Their inability to preserve structural information in noisy images highlights the limitations of non-learned methods for complex denoising tasks.

However, our model has certain limitations. It relies on large-scale labeled datasets for training, and its performance can vary with the diversity and quality of this data. To handle this problem, we use augmentation techniques to generate various images. The number of images increases from 780 to more than 2700, each 256*256*3. However, because the model has many complex layers that require massive GPU memory for handling images, the computational complexity during training and inference phases may pose challenges for real-time applications or resource-constrained environments.

To solve these problems, we divide input images into small patches (64*64*3) so the model is trained using more than 30,000 image patches. This makes the model faster and reduces GPU memory requirements, but this affects the model’s performance and requires more training images to reach its optimal accuracy. We try to find the optimal number and size of the patches needed to get the highest PSNR and SSIM values. Currently, we try to simplify the model by reducing the number and depth of layers without affecting the model’s performance.

While our model may have limitations, its key strength lies in its flexibility. It can be updated to handle various image enhancement problems, such as reducing noise or enhancing low-light images. We need to find a suitable dataset and update the loss function to find the best mapping between model evaluation metrics and loss function, thereby enhancing the model’s performance.

## Conclusion and future work

The proposed denoising algorithm effectively reduced speckle noise in medical images while preserving high perceptual quality in denoised images. The proposed model was evaluated using quantitative and qualitative metrics and compared with similar existing methods. The denoised medical images are evaluated using the quantitative measures PSNR and SSIM, where the proposed model shows significant improvements over the existing methods. Also, the qualitative assessment confirms visually appealing enhancements. The proposed model shows promising medical image enhancement results and outperforms similar methods.

Future work could optimize the proposed model to reduce computational complexity. The proposed model could be applied to denoise images in a low-light environment. Incorporating advanced transfer learning techniques with alternative cost functions could improve the model’s performance.

## Data Availability

The dataset analyzed during the current study is publicly available in the Kaggle repository at https://www.kaggle.com/datasets/aryashah2k/breast-ultrasound-images-dataset (accessed on 10 March 2025).

## Abbreviations

AE: Autoencoder
ADF: Anisotropic Diffusion Filtering
BN: Batch Normalization
CIDAE: Convolutional-based improved despeckling autoencoder
CNN: Convolutional Neural Networks
DA: Denoising Autoencoder
DAGAN: Deep De-Aliasing Generative Adversarial Networks
DCNND: Deep Convolutional Neural Network Denoising
DnCNN: Denoising Convolutional Neural Networks
DWT: Discrete Wavelet Transform
FFDNet: Fast and Flexible Denoising Convolutional Neural Network
GAD: Gradient Adaptive Denoising
GAN: Generative Adversarial Network
GAN-SRResNet: GAN for super-resolution and denoising
GAN-U-Net: Generative Adversarial Network with U-Net architecture
HTVM: Hybrid Total Variation Model
ID-CNN: Iterative Denoising CNN
INLSSM: Improved Non-Local Self-Similarity Method
IRCNN: Iterative Residual Convolutional Neural Network
MARU: Multi-scale Residual U-net
MK: Metric-Optimized Knowledge Distillation
NLM: Non-Local Means
OBNLM: Optimized Block Matching Non-Local Means
PCA-NLM: Principal Component Analysis with Non-Local Means
ReLU: Rectified Linear Unit
SBF: Statistical-Based Filtering
SNN: Speckle Noise Neural Network
PSNR: Peak Signal-to-Noise Ratio
SRAD: Speckle Reducing Anisotropic Diffusion
SRMD: Super-Resolution with Multiple Degradations
SSIM: Structural Similarity Index Measure
TV: Total Variation
UNLM: Unbiased Non-Local Means
WGAN-DUS: wavelet-based generative adversarial network for Despeckling ultrasound image
